# Non-invasive pressure-volume analysis: a novel method for evaluating ventricular function in patients with aortic stenosis

**DOI:** 10.3389/fcvm.2025.1740710

**Published:** 2026-01-22

**Authors:** Darijan Ribic, Espen W. Remme, Otto A. Smiseth, Richard J. Massey, Christian H. Eek, John-Peder Escobar Kvitting, Lars Gullestad, Kaspar Broch, Kristoffer Russell

**Affiliations:** 1 Department of Cardiology, Oslo University Hospital, Oslo, Norway; 2 Institute for Clinical Medicine, University of Oslo, Oslo, Norway; 3 Institute of Surgical Research, Oslo University Hospital, Oslo, Norway; 4 The Intervention Centre, Oslo University Hospital, Oslo, Norway; 5 Department of Cardiothoracic Surgery, Oslo University Hospital, Oslo, Norway

**Keywords:** aortic stenosis, load dependency, myocardial energetics, pressure-volume loops, ventricular function

## Abstract

**Background and aims:**

Conventional echocardiographic measurements like ejection fraction (EF) and global longitudinal strain (GLS) evaluate left ventricular (LV) function without considering concurrent loading conditions. A more comprehensive characterization of cardiac function and energetics can be achieved through pressure-volume analysis, but its clinical application is limited by the requirement for invasive measurements. We aimed to develop a clinically accessible, non-invasive method for pressure-volume loop analysis.

**Methods:**

We obtained simultaneous 3-dimensional echocardiograms and invasive LV pressures with micromanometer-tipped catheters during transcatheter aortic valve replacement (TAVR) for severe aortic stenosis. Volume-time traces from the echocardiograms were combined with invasive LV pressures and non-invasive pressure estimates to construct pressure-volume loops. We used echocardiograms before and after TAVR to evaluate changes in myocardial function via non-invasive pressure-volume studies.

**Results:**

In same-beat comparisons, stroke work calculated using non-invasive LV pressure estimations correlated well with stroke work calculated using invasive LV pressures (*r* = 0.95, ICC = 0.95, *p* < 0.0001, *y* = 0.90X + 1,836, mean bias −549 mmHg*mL, standard deviation 774 mmHg*mL; 95% limits of agreement: −2,006 to +967 mmHg*mL). After TAVR, stroke work fell substantially, ventricular efficiency increased, ventriculo-arterial coupling improved, and both total and resting energy consumption decreased. On the other hand, LV biplane EF and GLS remained unchanged.

**Conclusions:**

This study confirms the validity and clinical accessibility of non-invasive pressure-volume loop analysis in patients with aortic stenosis. The method identified and characterized changes in myocardial energetics, function, and ventriculo-arterial interaction, that are not typically detected by conventional echocardiography. These findings highlight the potential of non-invasive pressure-volume analysis in clinical and research practice.

## Graphical Abstract

**Figure:**
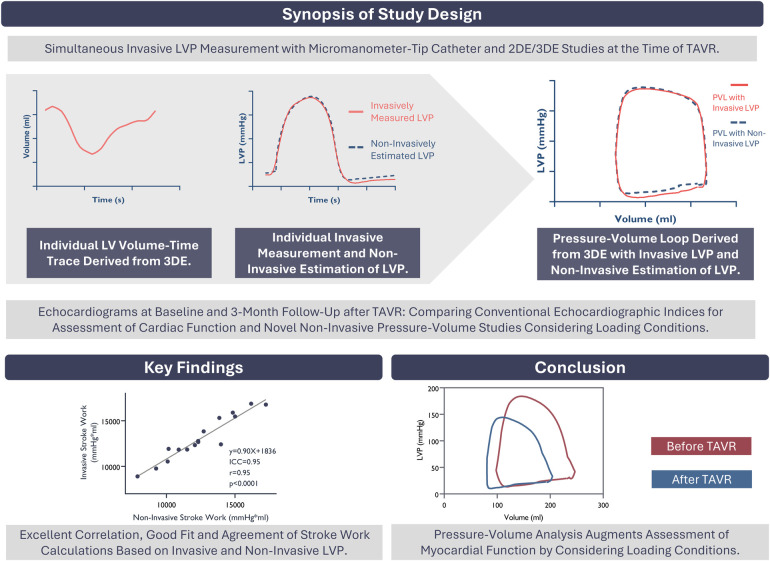
Study design. Key Findings. Conclusion. LVP, Left ventricular pressure; 2-3DE, two- and three-dimensional echocardiography; TAVR, transcatheter aortic valve replacement; PVL, pressure-volume loop; ICC, intra-class correlation coefficient.

## Introduction

Aortic stenosis (AS) is characterized by a progressive increase in left ventricular (LV) afterload, which leads to hypertrophic remodeling (1, 2). Simultaneously, contractile work and total oxygen consumption increase to maintain stroke volume (3). Conventional echocardiography has limited ability to assess the adaptive changes of LV function in this setting. Deformation indices used to assess systolic function and performance, such as ejection fraction (EF) and global longitudinal strain (GLS), are inherently load-dependent (4, 5) and do not account for concurrent loading conditions. Consequently, they fail to accurately reflect the increased myocardial work (6) and oxygen demand associated with elevated afterload. They also struggle to determine whether changes in measurements are due to alterations in myocardial properties or result from modifications in hemodynamics conditions. Therefore, there is a need for additional tools to assess LV function and energetics in the clinical setting while taking loading conditions into consideration.

Previously, we have shown that the myocardial work index (MWI) can be assessed non-invasively in patients with AS (7). This method uses pressure-strain loops to evaluate regional and global myocardial work. MWI accounts for afterload and reflects invasively determined myocardial work and oxygen demand (8). Notwithstanding, a significant limitation of the MWI is that it uses relative dimensions (strains), while physical work is based on absolute dimensions.

A more comprehensive understanding of myocardial function can be achieved through pressure-volume loop analysis (9). Here, concurrent ventricular loading conditions are integrated to characterize myocardial function and performance, while also providing insights into overall myocardial energy consumption (10). However, the construction of pressure-volume loops requires simultaneous invasive measurements of intraventricular pressure and volume (11), limiting its clinical use.

This study had two goals. First, we aimed to validate a novel, non-invasive method for pressure-volume analysis with potential for implementation in clinical practice. For this purpose, we used three-dimensional (3D) echocardiography and non-invasive LV pressure estimates (7, 8). We investigated the validity of this concept in patients with severe AS who had simultaneous invasive pressure measurements and echocardiography during transcatheter aortic valve replacement (TAVR). Second, we aimed to investigate the clinical feasibility of a fully non-invasive pressure-volume analysis in patients treated for severe AS. To achieve this goal, we obtained bedside echocardiograms at the time of admission for TAVR and again at 3-month follow-up.

## Material and methods

Among 20 patients with severe AS and preserved LV EF who participated in a study where we simultaneously obtained invasive pressure measurements and echocardiograms during TAVR (7), we included those with available and adequate 3D-echocardiography recordings. Patients with bicuspid valve or moderate or severe valvular disease other than aortic stenosis were excluded from the study. We acquired echocardiograms at admission for TAVR, during the procedure itself, and at a 3-month follow-up after TAVR. For validation purposes, invasive pressure measurements and echocardiograms were performed simultaneously, just prior to valve implantation during the TAVR procedure. Patients were screened to ensure that acceptable ultrasound image quality was achieved while they were in the supine position.

The Norwegian Regional Committee for Medical Research Ethics approved the study protocol. All subjects provided written informed consent.

### Invasive validation of pressure-volume estimates

#### Echocardiography and hemodynamic measurements

The periprocedural echocardiograms comprised apical two-dimensional (2D) views for speckle-tracking and 3D recordings for volume assessments (Vivid E95; GE Vingmed Ultrasound, Horten, Norway). Continuous-wave (CW) Doppler was used to assess aortic valve pressure gradients and to determine precise timing of aortic valvular events. Narrow-sector, zoomed 2D images acquired at optimized frame rates were used to determine the timing of mitral valve events. Invasive measurements of LV pressures were acquired with a micromanometer-tipped catheter (Millar Micro-Tip SPC-454F, Houston, TX, USA). Brachial blood pressure was measured with a sphygmomanometer while the patient was supine.

#### Non-invasive left ventricular pressure estimation

**Figure 1:**
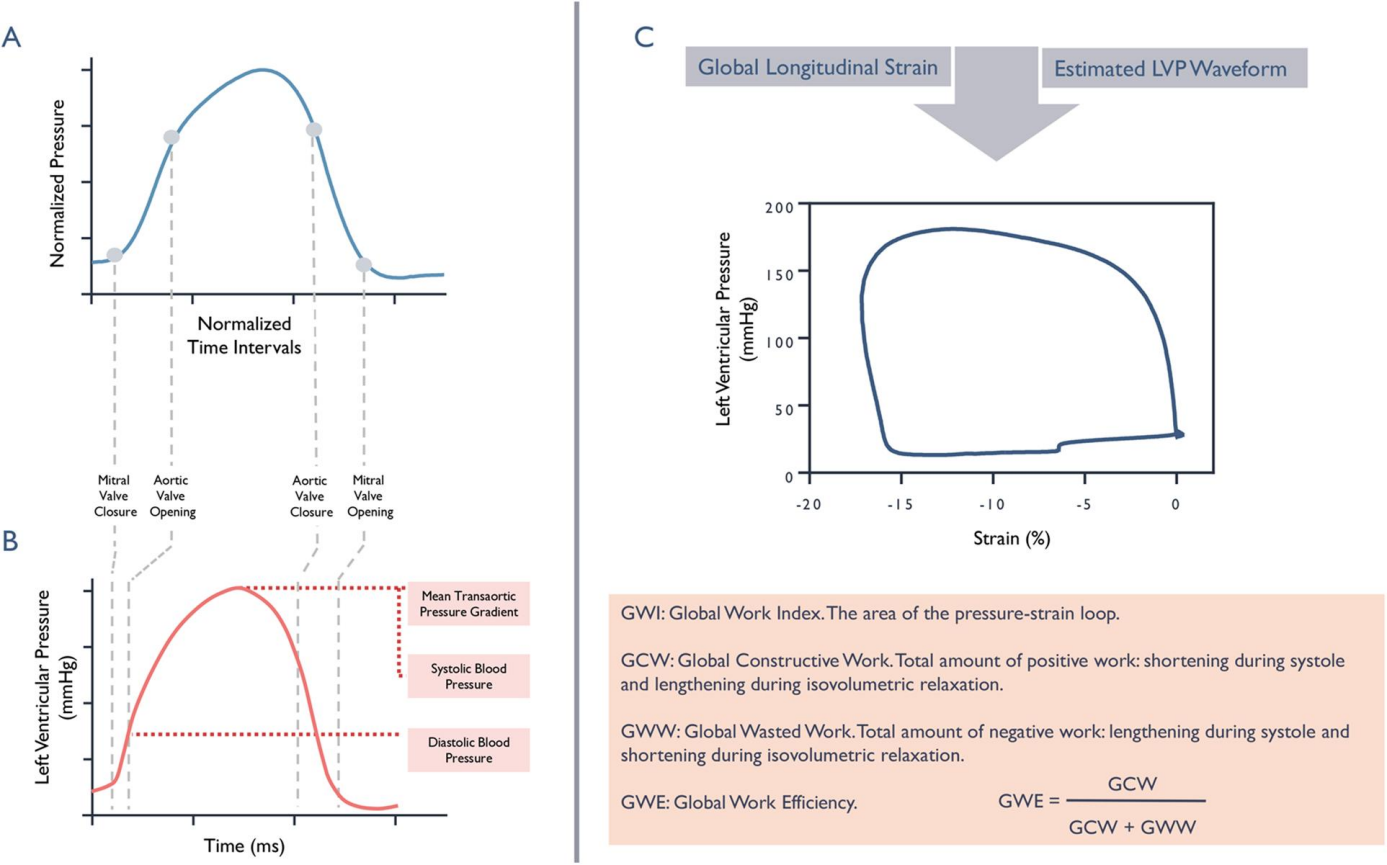
Non-invasive left ventricular pressure estimation and myocardial work calculations. Panels **A** and **B**: Schematic illustration depicting transition from a standardized reference curve (blue) to an estimated individualized left ventricular pressure waveform trace (red). Panel **C**: Myocardial work index, represented by the area of the pressure-strain loop and related indices.

The method for estimating individual LV pressure waveforms has been previously described and thoroughly validated for use in patients with and without aortic stenosis (7, 8). In brief, a generic reference LV pressure curve has been constructed by averaging and normalizing individual LV pressure tracings from patients with different heart conditions. This reference curve is scaled vertically and horizontally to coincide with estimated peak LV pressure and measured valvular events in the individual patient. For patients without significant outflow obstruction, LV peak pressure corresponds to the brachial systolic cuff pressure (8). For patients with AS, estimated LV peak pressure is the sum of the brachial systolic cuff pressure and the mean aortic transvalvular gradient (7, 12). Finally, the reference curve is adjusted so that the pressure at aortic valve opening equals the diastolic cuff pressure (7) (Figures 1A,B).

#### Pressure-volume loop analysis

**Figure 2:**
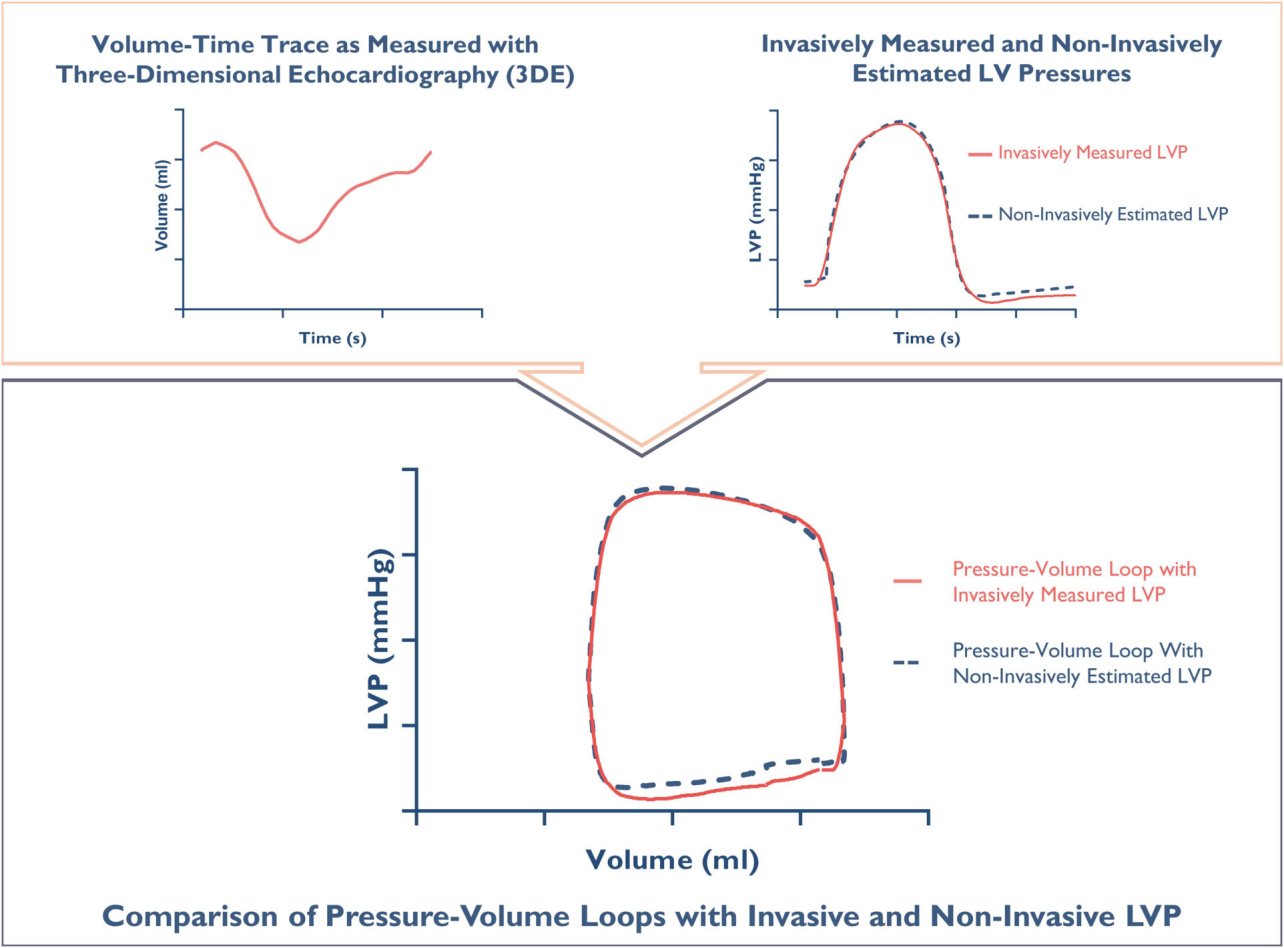
Constructing a pressure-volume loop. The upper panel: An individual volume-time trace measured with 3D-echocardiography, along simultaneously measured left ventricular pressure (LVP) (red) and non-invasive estimation of LVP (blue). The lower panel: Synchronized integration of pressures and volumes generates individual pressure-volume loops for direct same-beat comparison.

During the TAVR procedure, we simultaneously obtained shared recordings of LV volume using 3D-echocardiography and LV pressure through catheterization. The commercially available echocardiography analysis software EchoPAC (GE Vingmed Ultrasound, Horten, Norway) was utilized to measure LV volumes, allowing us to retrieve patient-specific LV volume-time traces that facilitated the construction of pressure-volume loops in this study. These measurements shared the same ECG trace, ensuring precise synchronization of LV volume and pressure. During the same periprocedural interval, brachial cuff pressure was measured using a sphygmomanometer. Mitral valvular events were assessed by 2D echocardiography, and gradients across the aortic valve together with the timing of aortic valvular were measured using CW Doppler. This allowed for the non-invasive estimation of LV pressure waveforms, as described above. By combining the LV volume-time traces with invasively measured and non-invasively estimated LV pressure waveforms, we created two sets of pressure-volume loops: one that incorporated invasively recorded LV pressures another that was fully non-invasive. Given that the invasive pressure measurements were done simultaneously with the 3D-echocardiograms and the brachial blood pressure measurement, same-beat comparisons of LV pressure–volume loops based on invasively measured LV pressure and non-invasive LV pressure estimates could be performed. This allowed us to evaluate the accuracy of non-invasive pressure–volume analysis (Figure 2).

From the pressure–volume loops, stroke work was estimated as the area enclosed by each loop. Estimate of ventricular contractility, end-systolic elastance, was obtained from the slope of the end-systolic pressure–volume relationship, defined as the line connecting the end-systolic pressure point on the loop to the volume axis at an arbitrary intercept V0, where V0 = 0 mL. The end-systolic point on each loop was identified as the point in the upper-left region of the loop with the greatest normalized distance from the loop's geometric center.

**Figure 3:**
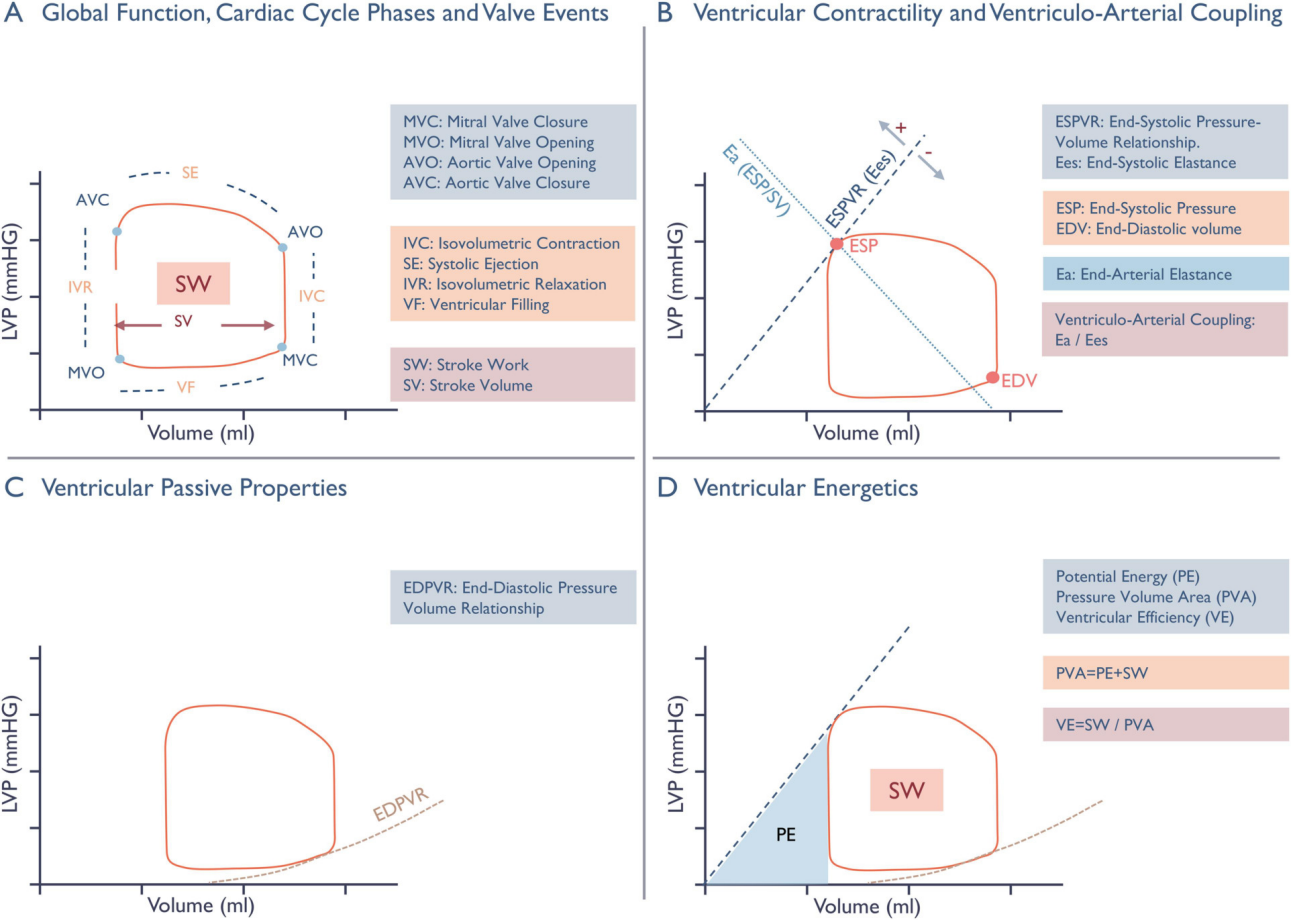
Schematic presentation of non-invasive pressure-volume analysis. Panel **A**: Cardiac cycle phases, valvular events, and *Stroke Volume* (SV) are illustrated on the pressure-volume (PV) loop, with the enclosed area representing *Stroke Work* (SW). Panel **B**: Ventricular contractility is expressed by the end-systolic elastance (Ees), the slope of the *end-systolic pressure-volume relationship* (ESPVR), and the one-beat contractility index ESP/EDV. The ESPVR line connects the point of *end-systolic pressure* (ESP) on the loop and the volume axis at an intercept (V0) where pressure equals to zero. V0 should ideally be found from multiple loops during preload constriction, but in our one-beat approach it is set to the origin. A leftward and upward shift of the ESPVR line enhances end-systolic elastance and the ESP/EDV ratio, reflecting an increase in contractility. The interaction between ventricular contractility and the arterial system, known as ventriculo-arterial coupling, is represented by the ratio of arterial elastance to end-systolic elastance. Arterial elastance serves as a surrogate measure of ventricular afterload, reflecting extra cardiac forces that oppose stroke volume ejection by relating end-systolic pressure to stroke volume. Panel **C**: Change in intraventricular pressure relative to volume during diastole defines the non-linear *end-diastolic pressure-volume relationship* (EDPVR), Characterizing passive mechanical properties through stiffness (Δ pressure/Δ volume) and compliance (Δ volume/Δ pressure). Panel **D**: The *pressure-volume area* (PVA) is the sum of *stroke work* and *potential energy (PE), representing* the total amount of mechanical energy generated during a cardiac cycle. Potential energy represents the energy stored in the ventricle at the end of systole, and is illustrated on the diagram by the area enclosed by the ESPVR line, the isovolumetric relaxation line, and the EDPVR line. It is directly influenced by the position of the PV-loop along and differentiates the various contributors to ventricular energy consumption, enabling expression of *ventricular efficiency* as the ratio of stroke volume and the total pressure-volume area.

We did not explicitly define an end-diastolic point on the loop because it was not incorporated in any specific calculations; however, end-diastolic volume, defined as the chamber's maximum volume, was used in several analyses. Ventricular afterload expressed as end-arterial elastance, calculated as the ratio of end-systolic pressure to stroke volume. End-diastolic volume was also used to compute single-beat contractility indices: the ratio of end-systolic pressure to end-diastolic volume and the ratio of maximum systolic pressure to end-diastolic volume. Ventriculo-arterial coupling, which describes the interaction between ventricular contractility and the arterial system, was expressed as the ratio end-arterial elastance to end-systolic elastance. Finally, ventricular efficiency was assessed by expressing stroke work relative to the total pressure–volume area (Figure 3).

### Ventricular function before and after TAVR

#### Non-invasive pressure-volume studies

At ***baseline*** (admission for TAVR), LV pressure waveforms were estimated while accounting for the mean transaortic pressure gradient in patients with severe AS. At the 3-month outpatient ***follow-up*** the LV pressure waveforms ware computed without including the transaortic gradient. These estimated pressure traces were then combined with corresponding baseline and follow-up LV volume traces in the same manner as the data acquired in the catheterization lab. This approach enabled us to create fully non-invasive, individualized pressure-volume loops for the patients at these two distinct time points.

#### Myocardial work index

Global myocardial work index, constructive and wasted work, and myocardial work efficiency were calculated by integrating LV pressure curves estimated non-invasively, as described earlier, with segmental strain traces obtained from 2D speckle-tracking GLS analysis using commercially available EchoPAC (GE Vingmed Ultrasound, Horten, Norway) (13) (Figure 1C).

### Statistical analysis

Values are expressed as mean ± standard deviation unless otherwise specified. We compared methods of measurement using least-squares linear regression, Pearson correlation coefficients, intra-class correlation coefficients (ICCs) with consistency of agreement based on a two-way mixed-effect model, and Bland–Altman plots with calculations of limits of agreement. Paired *t*-test was used for parametric data sets and the Wilcoxon rank-sum test for non-parametric data sets. Inter- and intraobserver variability for estimated non-invasive stroke work (the enclosed area of the pressure–volume loop) were assessed by reanalyzing ten randomly selected examinations from the clinical proof-of-concept cohort by two independent raters (A and B). Interobserver variability was evaluated using a two-way random-effects model, and intraobserver variability using a one-way random-effects model; agreement was also examined with Bland–Altman analysis.

Statistical analysis was performed with STATA SE 16.0 (StataCorp, College Station, TX, USA) and GraphPad Prism version 9.0.0 (Windows, GraphPad Software, Boston, Massachusetts USA, https://www.graphpad.com).

## Results

All of the 20 patients included in the original study (7) underwent echocardiography at admission as well as periprocedural echocardiography with simultaneous invasive intraventricular pressure recordings immediately before the TAVR. Follow-up echocardiograms were obtained in 19 patients 3.6 ± 0.7 months after the procedure. One patient declined evaluation due to the ongoing Covid-19 pandemic (Supplementary Figure 1). Determination of aortic and mitral valve events was feasible in all patients, as was successful non-invasive estimation of the LV pressure waveforms.

### Invasive validation of pressure-volume studies

Successful recordings of LV volume-time traces and simultaneous invasive pressure measurements, obtained immediately before the implantation of the aortic valve prosthesis and deemed adequate for further analysis, were available for 16 of the 20 study subjects. These 16 patients were all in sinus rhythm and constituted our ***validation cohort***.

We used multi-beat real-time 3D-echocardiography to obtain LV time-volume traces. In four patients, the image frame rate was below 12 frames per s, which hindered the successful generation of volume-time traces. In the remaining 16 patients, the average frame rate for the 3D LV studies was 31 ± 0 frames per s. The validity of the non-invasive pressure-volume analysis was tested by same beat comparison in these 16 patients.

**Figure 4:**
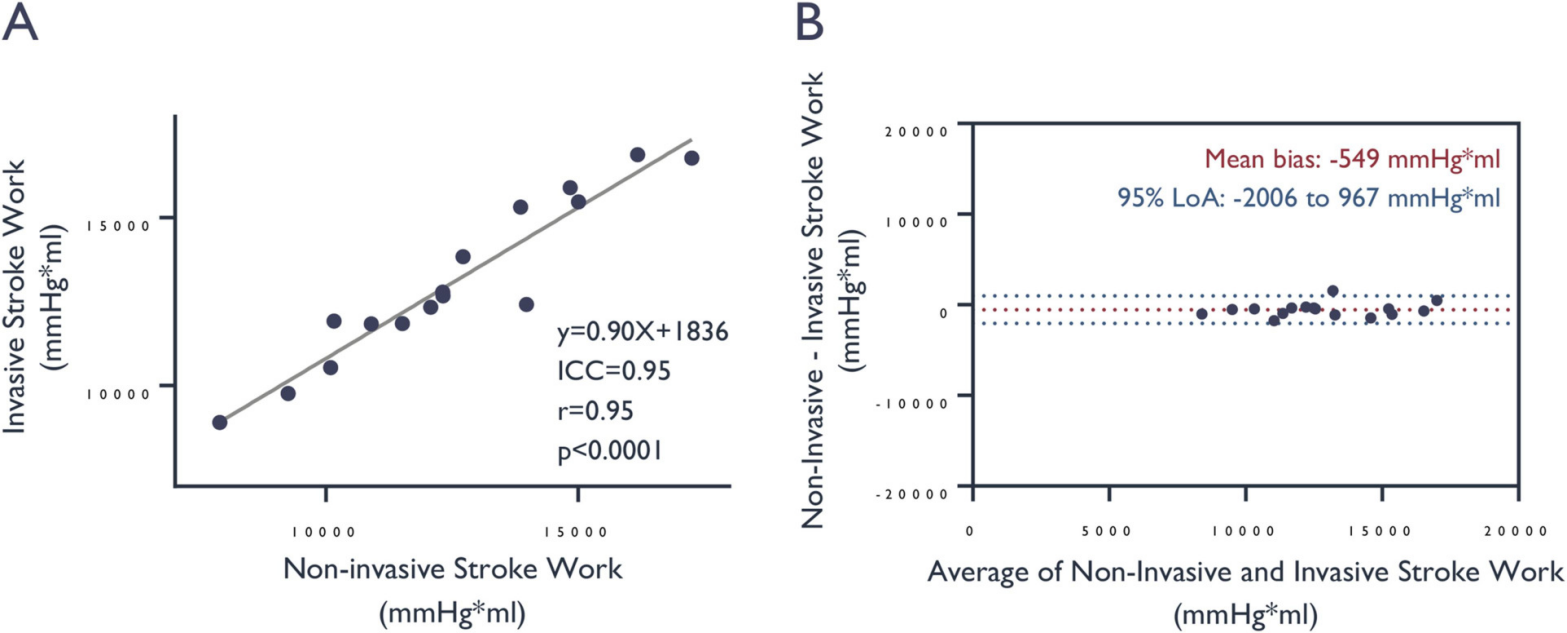
Comparison of stroke work values derived from invasive vs. non-invasive LV pressure assessment. **(A)** Correlation and **(B)** agreement of stroke work, determined by the area enclosed within the pressure-volume loop, based on measured *invasive* and *estimated* left ventricular pressures. ICC, intra-class correlation coefficient.

The fully non-invasive stroke work estimates, expressed as the area enclosed by the pressure-volume loop constructed from 3D-echocardiograms and non-invasively estimated LV pressure corresponded well with the stroke work calculated by invasively measured LV pressure. There was excellent correlation, good fit (*r* = 0.95, ICC = 0.95, *y* = 0.90*X* + 1,836, *p* < 0.0001) between the stroke work as appraised by the two methods with a strong agreement relative to the mean stroke work of 13,070 ± 2,417 mmHg*mL: mean bias −549 mmHg*mL, standard deviation 774 mmHg*mL; 95% limits of agreement: −2,006 to +967 mmHg*mL (Figure 4).

**Figure 5:**
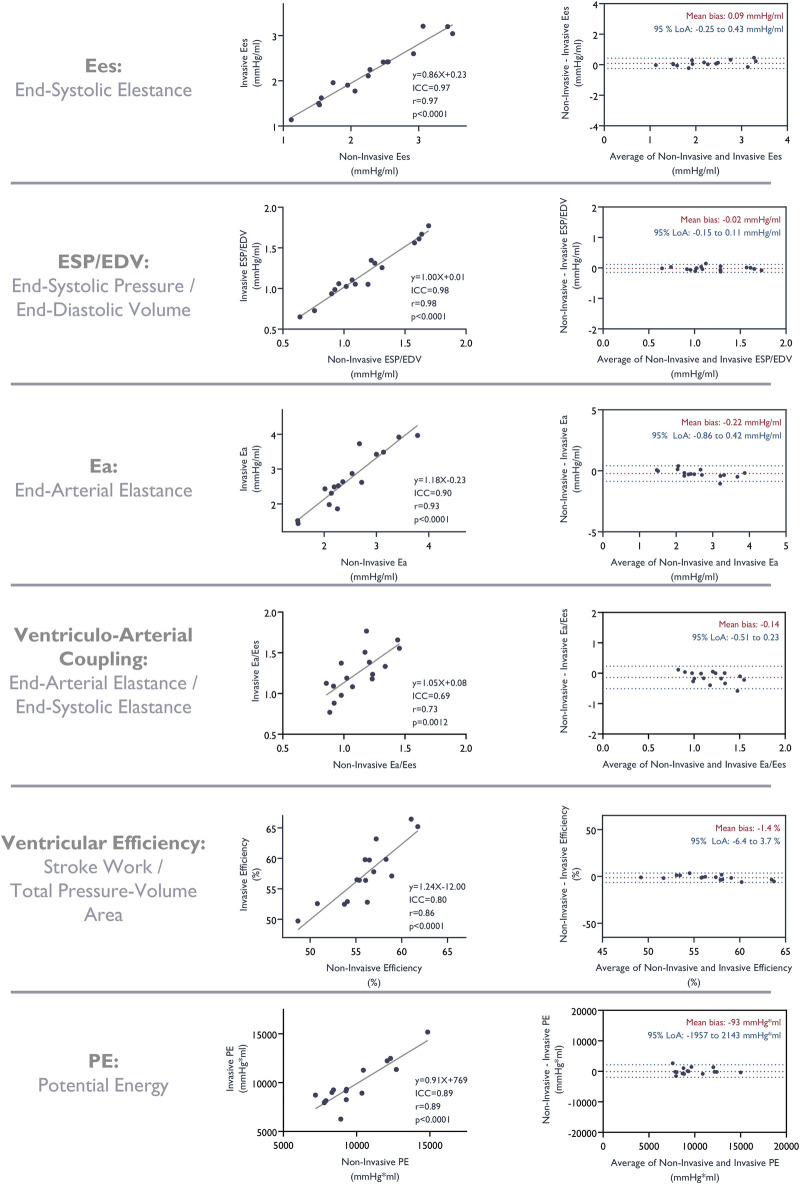
Correlation and agreement of metrics derived from the pressure-volume analysis based on invasive measurements and non-invasive estimates of LV pressures. ICC, intra-class correlation coefficient.

Similarly, we observed good correlation and a good fit with strong agreement between parameters derived from pressure-volume loops constructed from non-invasive LV pressure estimates and those based on invasively measured pressures. LV end-systolic elastance, end-arterial elastance, ventricular efficiency, ventriculo-arterial coupling, and potential energy as assessed by pressure-volume loops based on estimated pressures all correlated strongly with those based on invasive measurements (Figure 5).

### Ventricular function before and after the TAVR procedure

Successful paired non-invasive pressure-volume analysis was achieved at the time of admission and at the 3-month follow-up in 17 of the 20 patients; all 17 were in sinus rhythm. These analyses formed our ***clinical proof-of-concept cohort*** and allowed for further investigation of potential changes in ventricular properties associated with TAVR. Table 1 presents baseline demographic characteristics for this cohort. Volume analysis by 3D-echocardiography was not feasible in one patient at baseline and in two patients at follow-up due to technical issues related to heartbeat variability at the time of examination. Additionally, one patient was lost to follow-up, which has already been noted.

**Table 1:** Baseline characteristics (*n* = 17).

Variable	
Sex (*n*,%)	
Female	10 (59%)
Age (years)	77 ± 5
Body mass index (kg/m^2^)	26.4 ± 4.4
Hypertension (*n*,%)	16 (94%)
Coronary artery disease (*n*,%)	11 (65%)
Diabetes mellitus (*n*,%)	2 (12%)
Systolic blood pressure (mmHg)	159 ± 28
Diastolic blood pressure (mmHg)	73 ± 12

Values are reported as mean ± standard deviation for continuous variables, and *n* (%) for categorical variables.

Calculation of the MWI was feasible in all patients who underwent conventional 2D-echocardiography and Doppler measurements at baseline (*n* = 20) and at follow up (*n* = 19). To compare the conventional echocardiographic and MWI indices with parameters derived from non-invasive pressure-volume analysis, we excluded data from patients who did not have paired non-invasive pressure-volume measurements at both baseline and follow-up. Thus, we present data for the 17 patients with paired datasets at both time points.

**Figure 6:**
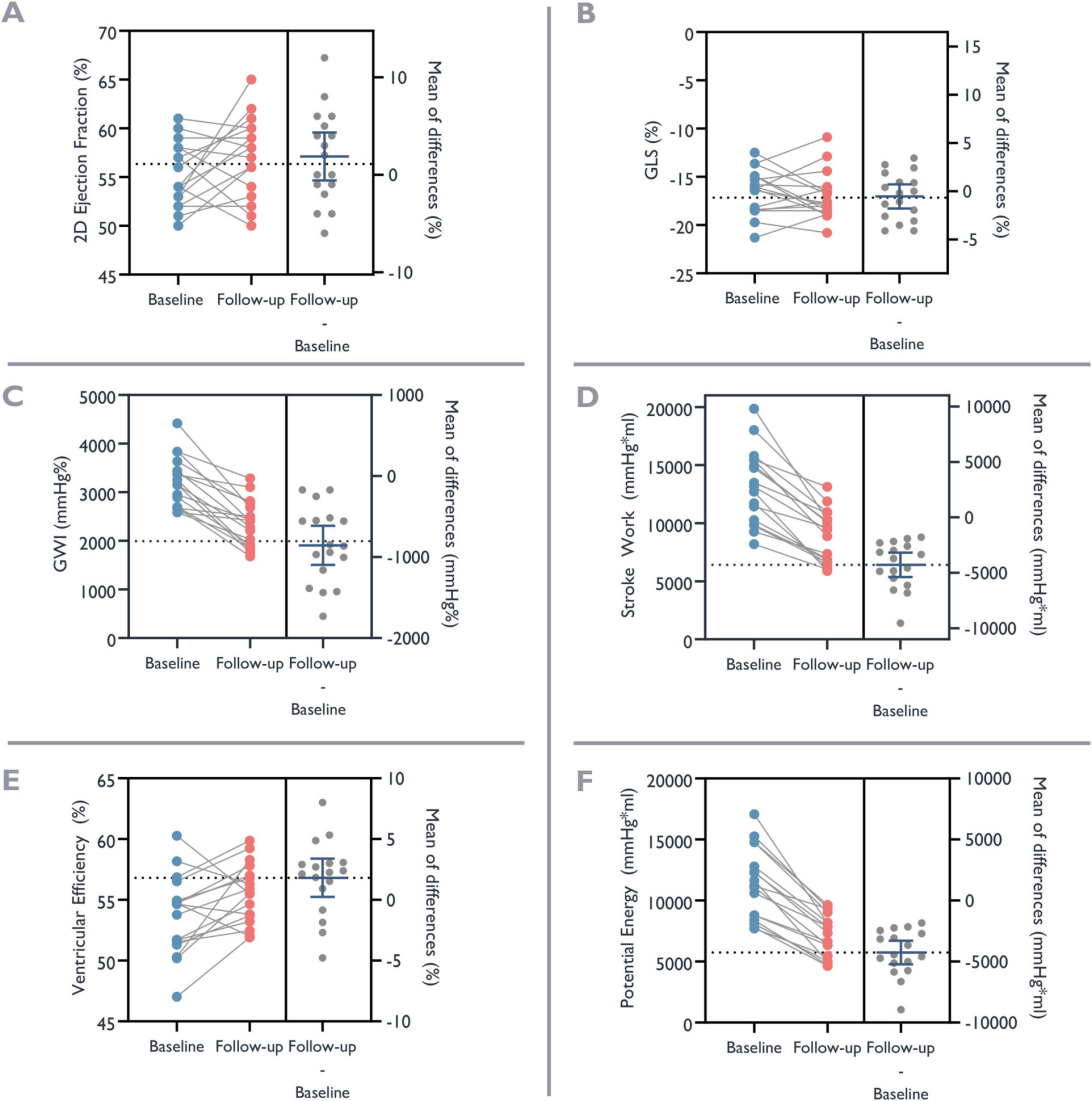
Ventricular function before and after valve replacement. Baseline ventricular function at the time of admission for valve replacement (blue dots) and at follow-up after valve replacement (red dots). The left panel of each graph depicts trend of change, and the right panel the individual and mean differences. **(A)** Ejection Fraction with 2D-echocardiography. **(B)** Global Longitudinal Strain (GLS). **(C)** Global Work Index (GWI). **(D)** Stroke Work. **(E)** Ventricular Efficiency. **(F)** Potential Energy.

The results of conventional echocardiograms, MWI, and non-invasive pressure-volume studies before and following TAVR are presented in Table 2 and Figure 6. The systolic blood pressure was 159 ± 28 mmHg at baseline compared to 159 ± 17 mmHg at follow-up, with mean difference 0 ± 25 mmHg, (*p* = 0.96). While there was no significant change in mass or end-diastolic intracavitary diameter, there was a significant reduction of systolic and diastolic volumes from baseline to follow-up. Systolic function, as assessed by 3D EF, improved slightly. Notably, there were no statistically significant changes in 2D EF or GLS. In contrast, we observed substantial changes for myocardial work indices as well as for metrics derived from pressure-volume loops. We observed a reduction of −855 mmHg% ± 471 mmHg% and −854 mmHg% ± 582 mmHg% for MWI and constructive myocardial work, respectively. There was also a substantial and significant reductions in stroke work of −4,280 mmHg*mL ± 2,124 mmHg*mL, and in potential energy −4,256 mmHg*mL ± 1,886 mmHg*mL. Furthermore, there was a significant reduction in end-arterial elastance expressing afterload (mean difference—0.4 ± 0.3), and contractility expressed by the ratio of end-systolic pressure to end-diastolic volume (mean difference—0.1 ± 0.1). End-systolic elastance also decreased, but the change was not statistically significant. Ventriculo-arterial coupling improved, with a relative reduction of 13%, indicating a more optimal interaction between the ventricle and arterial system, and ventricular efficiency improved by 1.8% ± 3.1%. Both changes were statistically significant.

**Table 2:** Conventional and novel echocardiographic parameters at admission and follow-up (*n* = 17).

Conventional echocardiographic parameters	Baseline	Follow-up	*P*-value
LV end-diastolic diameter (cm)	4.7 ± 0.6	4.6 ± 0.5	0.540
LV mass index 2D (g/m2)	84 ± 23	76 ± 17	0.083
LV end-diastolic volume 2D (mL)	170 ± 41	148 ± 35	<0.001
LV end-systolic volume 2D (mL)	77 ± 21	63 ± 15	0.001
LV end-diastolic volume 3D (mL)	172 ± 40	148 ± 36	<0.001
LV end-systolic volume 3D (mL)	79 ± 20	64 ± 17	<0.001
LV stroke volume 3D (mL)	93 ± 23	84 ± 22	0.002
LV ejection fraction 2D (%)	55 ± 3	57 ± 4	0.123
LV ejection fraction 3D (%)	54 ± 4	57 ± 5	0.029
Aortic valve velocity (m/s)	4.6 ± 0.5	2.2 ± 0.5	<0.001
Aortic transvalvular mean gradient (mmHg)	51 ± 13	11 ± 5	<0.001
Heart rate (beats per min)	67 ± 18	64 ± 10	0.854
Global longitudinal strain (%)	16.5 ± 2.3	17.0 ± 2.4	0.369
Myocardial work index parameters	Baseline	Follow-up	*P*-value
Global work index (mmHg%)	3,208 ± 519	2,353 ± 475	<0.001
Global constructive work (mmHg%)	3,549 ± 619	2,696 ± 469	<0.001
Global wasted work (mmHg%)	128 ± 58	132 ± 74	0.859
Global work efficiency (%)	96.0 ± 1.8	94.6 ± 3.0	0.125
Pressure-volume analysis parameters	Baseline	Follow-up	*P*-value
Stroke Work (mmHg*mL)	13,182 ± 3,234	8,902 ± 2,249	<0.001
End-Systolic Elastance (mmHg/mL)	2.36 ± 0.75	2.22 ± 0.60	0.099
Arterial Elastance (mmHg/mL)	2.65 ± 0.63	2.22 ± 0.60	<0.001
Ventricular Efficiency (%)	53.7 ± 3.3	55.5 ± 2.5	0.027
End-Systolic Pressure/End-Diastolic Volume (mmHg/mL)	1.24 ± 0.32	1.10 ± 0.28	<0.001
Maximal Systolic Pressure/End-Diastolic Volume (mmHg/mL)	1.28 ± 0.33	1.13 ± 0.29	<0.001
Ventriculo-Arterial Coupling	1.17 ± 0.22	1.01 ± 0.18	0.013
Pressure Volume Area (mmHg*mL)	24,556 ± 5,950	16,020 ± 3,915	<0.001
Potential Energy (mmHg*mL)	11,374 ± 2,900	7,118 ± 1,743	<0.001

Values are reported as mean ± standard deviation. LV, left ventricular.

### Interobserver and intraobserver variability

Both inter- and intraobserver comparisons of stroke work measurements showed strong agreement. Mean biases were −420 mmHg*mL and −102 mmHg*mL, respectively, relative to the mean estimated stroke work of 13 172 mmHg*mL. Intraclass correlation coefficients were high for both interobserver and intraobserver variability (ICC = 0.97 for each) (Supplementary Figure 2).

## Discussion

This validation and proof-of-concept study shows that non-invasive pressure-volume analysis based on 3D-echocardiography and non-invasive LV pressure estimates is feasible and attainable in patients with severe AS. We demonstrate that non-invasive pressure-volume loops and their derived parameters correlate and agree well with values obtained from invasive pressure measurements. Our findings are consistent with those of a recent study that investigated non-invasive pressure-volume analysis in an experimental animal model (14).

To our knowledge, this is the first study to directly compare beat-to-beat pressure-volume loops obtained non-invasively to pressure-volume loops derived from invasive pressures in patients with severe AS. Furthermore, our results highlight the inability of conventional deformation indexes to accurately reflect changes in LV performance under different loading conditions.

**Figure 7:**
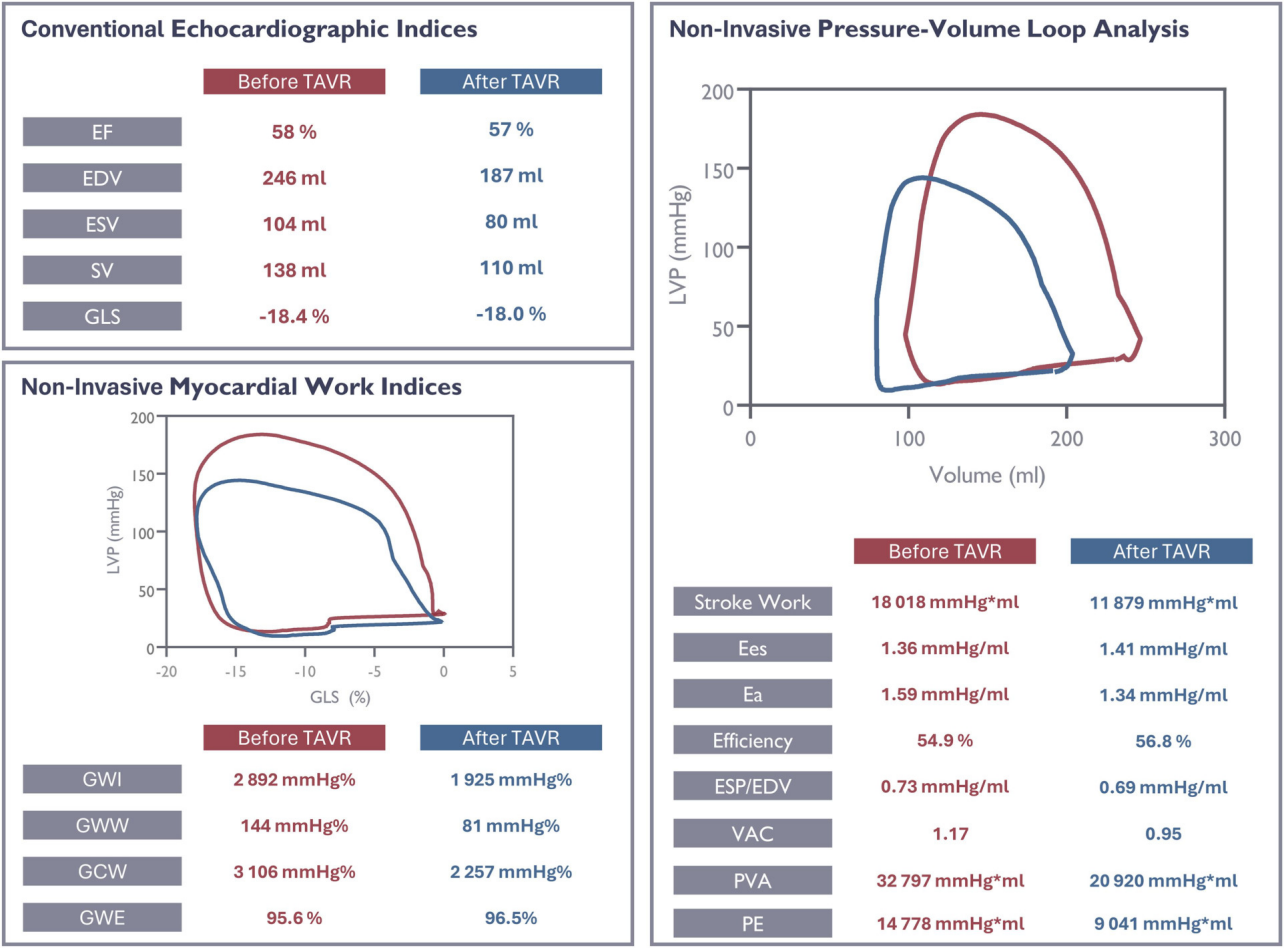
Evaluation of ventricular function in a study patient using conventional and novel echocardiographic parameters before and after valve replacement. EF, ejection fraction; EDV, end- diastolic volume; ESV, end-systolic volume; SV, stroke volume; GLS, global longitudinal strain; GWI, global work index; GWW, global wasted work; GCW, global constructive work; GWE, global work efficiency; Ees, end-systolic elastance; Ea, end-arterial elastance; VE, ventricular efficiency; VAC, ventriculo-arterial coupling; PVA, pressure-volume area; PE, potential energy.

We investigated a small cohort of patients with severe AS. On average, the patients had slightly elevated LV volumes, normal LV internal dimensions, normal LV mass index and preserved systolic function. In these patients, we observed that the left ventricle effectively compensates for the increased afterload. As expected, and as previously reported TAVR resulted in substantial reductions in transaortic mean valve gradients, but also in a matched reduction of diastolic and systolic LV volumes (15, 16). Reduction of LV volumes without substantial changes in intraventricular dimensions has previously been observed in patients undergoing surgical treatment for severe AS (17). Importantly, we did not detect any significant changes in 2D LV EF and GLS, while 3D LV EF showed a slight improvement. The systolic blood pressure remained unchanged. Interestingly, in our cohort, the benefits of outflow obstruction relief after TAVR were undetectable by conventional methods that are widely used to evaluate systolic function (Figure 7).

Conversely, significant changes were noted in several parameters derived from pressure-volume studies and the MWI when accounting for variations in loading conditions. After TAVR we observed statistically significant reductions in global total myocardial work and global constructive myocardial work. These findings primarily reflect the consequences of reduced workload due to the decreased afterload after valve implantation. As previously reported, the decrease in total myocardial work and constructive myocardial work did not result in significant changes in the quantity of wasted work or work efficiency (12), thus reflecting a synchronous deformation pattern (by strain) following TAVR.

The non-invasive MWI uses segmental GLS traces and LV pressure estimations to quantify regional and global myocardial work. In contrast, the non-invasive pressure-volume loop analysis incorporates intraventricular pressures and the total volume change throughout the cardiac cycle. Thus, replacing strain, a relative measure, by absolute volume. In this regard, pressure-volume analysis provides absolute measures of global LV properties, overcoming the limitations of a relative measure such as strain. This has the potential to impact on the stroke work values obtained from the two methods. For example, a dilated compensated ventricle with maintained stroke volume, but significantly reduced strain, will have a severely reduced MWI, whereas absolute values obtained from pressure-volume analysis reflect the preserved ability to provide an adequate cardiac output. The two methods illuminate different aspects of compensatory mechanisms, global and regional properties, and pattern of myocardial deformation. Hence, they have the potential to complement each other for a comprehensive evaluation of myocardial performance. This is underscored by the following observation: after TAVR, there was a greater relative reduction in stroke work, as determined by pressure-volume loop analysis, compared to the reduction in the MWI as assessed by pressure-strain loop analysis: 32% vs. 26%, respectively. Although the two methods demonstrated consistent change, the associated LV volume change impacted the two work estimates differently, contributing to a difference in the relative change of work estimates.

Following TAVR, the parameters derived from pressure-volume analysis were directly influenced by the reduction of outflow obstruction as well as by the change in LV volumes. While there were no significant changes in EF and GLS, all but one of the pressure-volume parameters changed significantly after TAVR, underscoring the sensitivity of pressure-volume analysis to detect dynamic changes in LV properties. Previous studies, conducted soon after TAVR in patients with preserved EF, have found only marginal changes in EF and GLS (18). Our cohort was small, and we acknowledge that this may limit the ability to detect modest changes for EF and GLS. However, it is important to contrast these findings with the substantial changes in parameters that account for loading variations within the same cohort.

In patients treated with TAVR, conventional echocardiographic evaluation revealed a reduction of transaortic velocities, gradients, and LV end-systolic/diastolic volumes, while conventional deformation indices used for assessing systolic function remained unchanged. The 3D LV EF assessment picked up a slight improvement in systolic function. However, changes observed after TAVR were more pronounced when integrating concurrent loading conditions. After TAVR, we observed a significant reduction in global and constructive myocardial work and a reduction in stroke work, as derived from pressure-volume studies. Furthermore, we were able to document effects of the TAVR procedure, evident by a reduction in contractile load with a reduction of end-systolic pressure to end-diastolic volume ratio and optimalisation of the ventriculo-arterial coupling (19) with an increase in ventricular efficiency, all while reducing potential energy. End-systolic elastance also declined; however, this decline did not reach statistical significance. The pressure-volume studies were able not only to document a reduction in cardiac work during systole, as is the case for myocardial work indices, but also shed light on important aspects regarding the resting state of the ventricle and the interaction between the ventricle and the arterial system. Figure 7 illustrates these observations at the individual level. It is important to note that the blood pressure remained unchanged after TAVR, suggesting that the primary drivers of change in loading conditions were the reduction in transvalvular gradient and the subsequent alterations in ventricular volume. Further studies should assess these metrics in a larger cohort of patients under varying degrees of afterload to investigate whether cumulative workload and ventricular adaptation under increasing afterload are related to the accumulation of myocardial fibrosis, and to determine how this relationship influences prognosis. In turn, these findings could inform clinical decision-making.

### Accessibility of the method

As is true for echocardiography in general, the quality of the ultrasound image is crucial for accurate assessment of LV volumes with 3D-echocardiography and the subsequent non-invasive pressure-volume analysis. In our study, the ability to acquire adequate 3D-volume studies in some patients was limited not by image quality, but by inadequate frame rates during the examination and by variations in RR-interval. Both issues are technical and can be overcome by awareness and understanding of the method.

Over the past two decades, 3D-echocardiography has become increasingly accessible as a bedside tool for assessing LV volumes and function. Compared with 2D-echocardiography, 3D-echocardiography provides results that are more reliable and correlate better with those obtained by cardiac magnetic resonance imaging, the gold standard for cardiac volume measurements (20). Unlike 2D-echocardiography, 3D-echocardiography does not rely on geometric assumptions about LV shape and avoids foreshortening, while achieving acceptable intra- and interobserver reproducibility (20).

The method for estimating non-invasive LV pressures was successful in all study subjects and the validity of this approach is well-established (7, 8). Our validation cohort consisted of patients with severe AS. However, the method for non-invasive pressure-volume loop analysis, as outlined in this study, can also be applied to patients without outflow obstruction. In such cases, the estimation of LV pressure relies solely on the brachial systolic blood pressure, without the need to incorporate the mean trans-aortic pressure gradient.

The described method is based on data that are readily available using commercial software, and it showed strong correlation and agreement with respect to inter- and intraobserver variability. This suggests that the integration of non-invasive pressure estimates and volumes could be streamlined to become an integral part of the routine LV work-up.

## Limitations

Non-invasive pressure-volume loop analysis as proposed herein is a clinically oriented single-beat method of calculating specific ventricular metrics that incorporate actual loading conditions. The method offers parameter estimates derived from pressure-volume loops that differ from those obtained through traditional invasive pressure-volume studies. Here, multiple recordings under varying loading conditions are recorded and analyzed to determine contractile and passive properties of the left ventricle. This is neither possible nor practical in the bedside setting. Furthermore, the method does not take heart rate into account: therefore, changes in volume and absolute values derived from pressure-volume studies must be evaluated with this in mind. The method operates with absolute values that require normalization for comparison between individual patients. As previously documented (7), our method for estimating LV pressure tends to overestimate filling pressures when LV systolic pressure is substantially elevated. Acknowledging the model's limited accuracy during the filling phase, we therefore did not assess the end-diastolic pressure–volume relationship in this study. In patients with very high systolic pressure, the model also underestimates pressure–volume loop area relative to true values resulting in an underestimation of stroke work. This discrepancy is less pronounced at normal LV pressures.

We have arbitrarily set the V0, the theoretical LV volume at zero pressure, to zero. Therefore, our definition of the end-systolic pressure-volume relationship differs from the classical assumption. This influences the calculations of ventriculo-arterial coupling, end-systolic elastance, potential energy and ventricular efficiency. True V0 will always be a positive, finite volume. Hence, the non-invasive end-systolic elastance value with V0 = 0 mL will be lower compared to its true value.

## Conclusion

This study confirms the feasibility of a non-invasive method of pressure-volume loop analysis using concurrent 3D-echocardiography LV volumes and non-invasive LV pressure estimations. Non-invasive pressure-volume loop analysis enhances the understanding of the effects of TAVR on the change in global stroke work, ventricular energetics and ventriculo-arterial coupling and may aid clinicians in the assessment of patients with AS.

## Data Availability

The raw data supporting the conclusions of this article will be made available by the authors, without undue reservation.

## Supplementary material

Supplementary Figure 1 Study Cohort.

Supplementary Figure 2 **Inter- and intraobserver variability.** A. Scatter plot comparing estimated Stroke Work (mmHg*mL) measured by examiners A (A1) and B (B1), with the linear regression equation, intraclass correlation coefficient (ICC), and Pearson's correlation coefficient (r). B. Scatter plot comparing estimated Stroke Work (mmHg*mL) measured by examiner A at two time points (A1 and A2), with the linear regression equation, ICC, and Pearson's r. C. Bland-Altman plot comparing estimated Stroke Work (mmHg*mL) measured by examiners A (A1) and B (B1), showing the mean bias (mean difference) and the 95% limits of agreement. D. Bland-Altman plot comparing estimated Stroke Work (mmHg*mL) measured by examiner A at two time points (A1 and A2), showing the mean bias and the 95% limits of agreement.
