# Supplementary figures and images for: Non-invasive pressure-volume analysis: a novel method for evaluating ventricular function in patients with aortic stenosis
Source: Front Cardiovasc Med. 2026 Jan 22;12:1740710. doi: 10.3389/fcvm.2025.1740710 (PMC12872862; doi:10.3389/fcvm.2025.1740710)

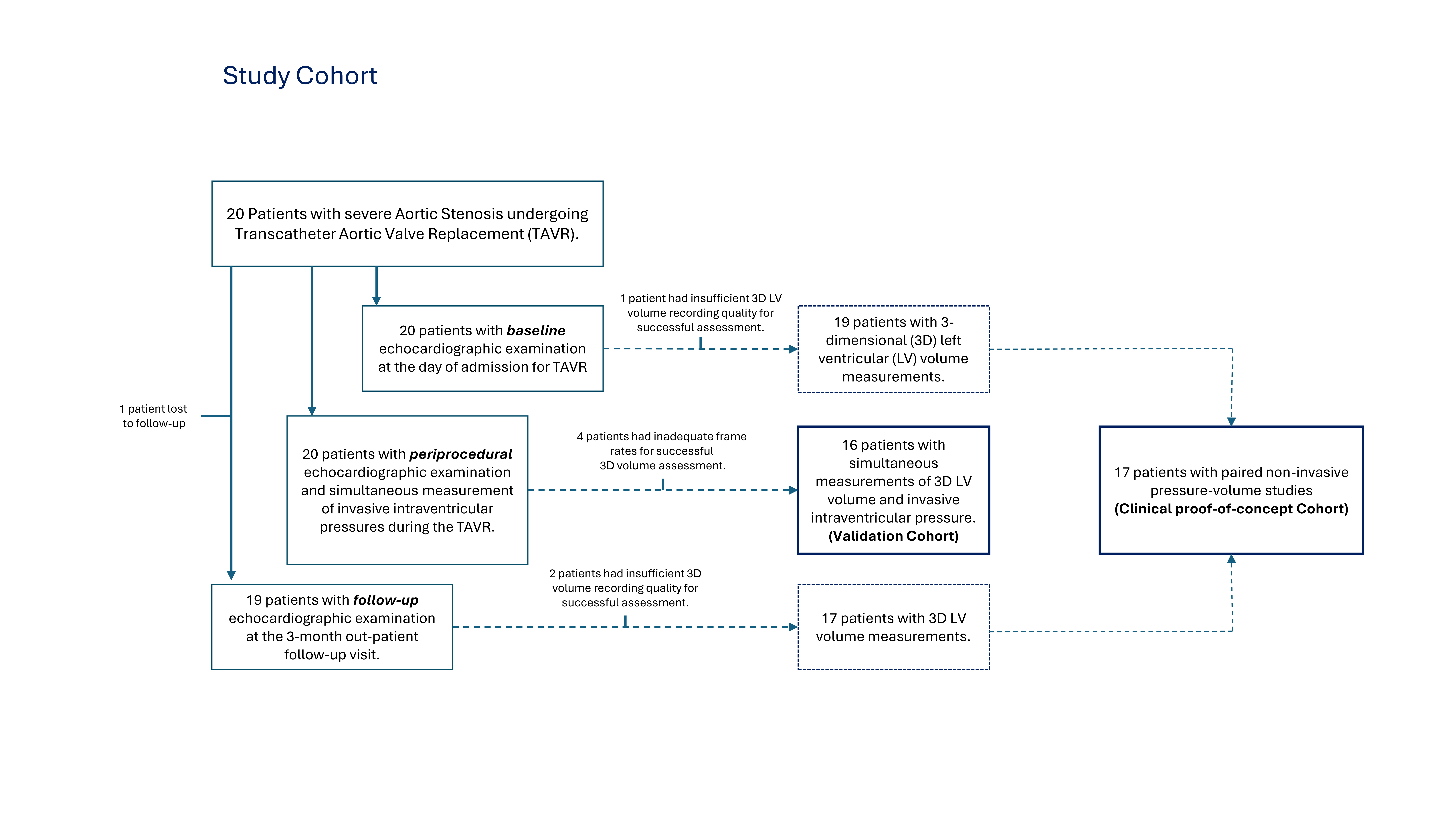

Supplement: Supplementary Figure 1 — Study Cohort. [file Image1.tif]

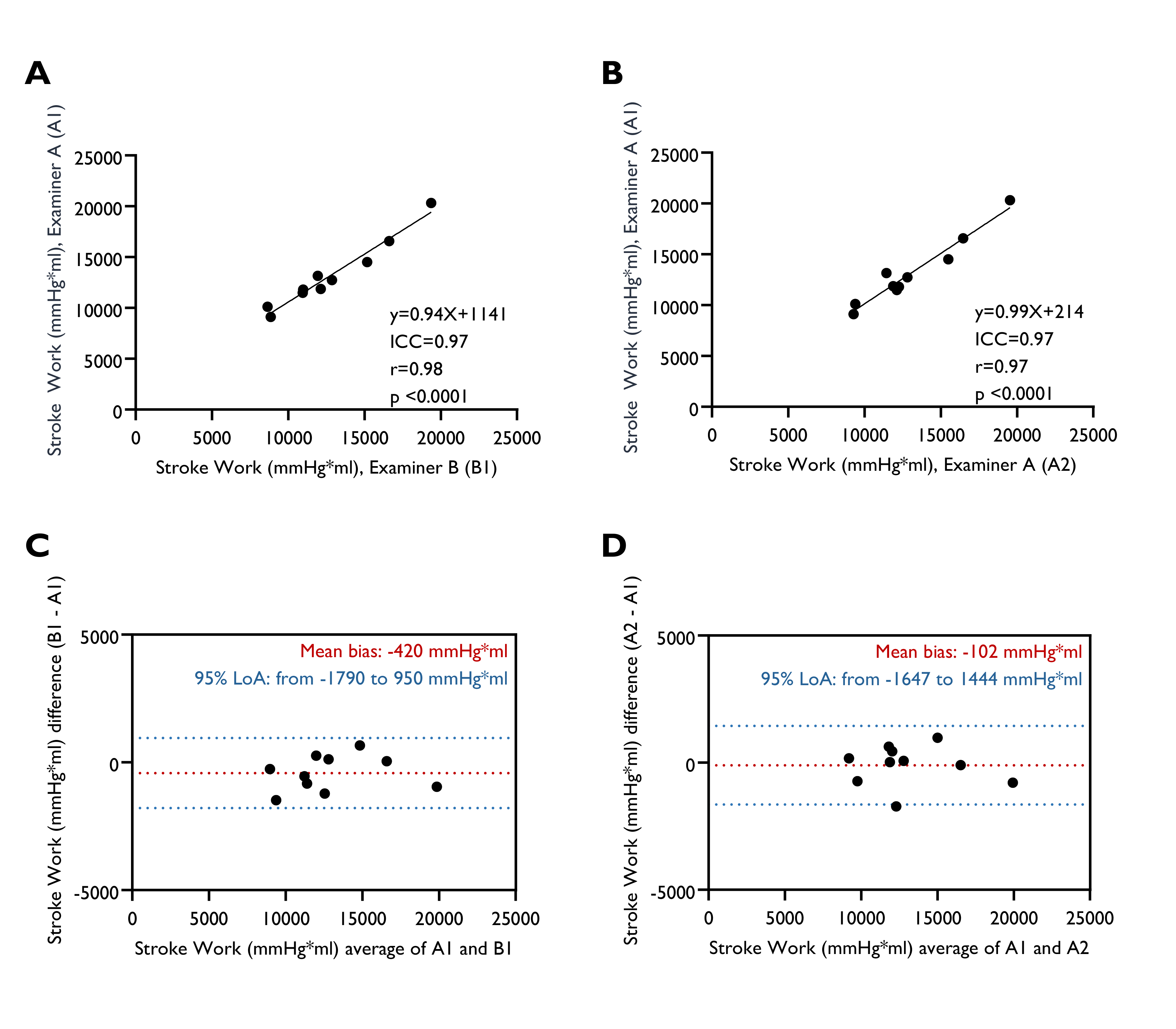

Supplement: Supplementary Figure 2 — Inter- and intraobserver variability. A. Scatter plot comparing estimated Stroke Work (mmHg*mL) measured by examiners A (A1) and B (B1), with the linear regression equation, intraclass correlation coefficient (ICC), and Pearson's correlation coefficient (r). B. Scatter plot comparing estimated Stroke Work (mmHg*mL) measured by examiner A at two time points (A1 and A2), with the linear regression equation, ICC, and Pearson's r. C. Bland-Altman plot comparing estimated Stroke Work (mmHg*mL) measured by examiners A (A1) and B (B1), showing the mean bias (mean difference) and the 95% limits of agreement. D. Bland-Altman plot comparing estimated Stroke Work (mmHg*mL) measured by examiner A at two time points (A1 and A2), showing the mean bias and the 95% limits of agreement. [file Image2.tif]
